# Superior Mesenteric Artery Thrombosis and Intestinal Ischemia as a Consequence of COVID-19 Infection

**DOI:** 10.7759/cureus.37259

**Published:** 2023-04-07

**Authors:** Ashik Pokharel, Indira Acharya, Ranjit K Chaudhary, Swachchhanda Songmen, Richard Williams

**Affiliations:** 1 Internal Medicine, MedStar Union Memorial Hospital, Baltimore, USA; 2 Internal Medicine, MedStar Georgetown University Hospital, Washington DC, USA; 3 Radiology, St. Vincent's Hospital, Bridgeport, USA; 4 Radiology, St. Vincent's Medical Center, Bridgeport, USA; 5 Gastroenterology and Hepatology, MedStar Georgetown University Hospital, Washington DC, USA

**Keywords:** superior mesentery artery (sma), morbidity, multifactorial, thrombotic events, covid-19

## Abstract

COVID-19-associated arterial and venous thrombotic events are multifactorial in origin, resulting in significant morbidity and mortality. Intestinal ischemia due to thrombus is a rare manifestation of COVID infection. Here, we report the case of a patient who presented with fever, malaise, and diarrhea, and was found to be COVID-19 positive; his clinical course was further complicated by devastating thrombosis of the superior mesentery artery (SMA) associated with COVID-19 infection.

## Introduction

The development of thrombosis in COVID-19 is caused by a combination of factors. These include ongoing inflammation due to infection and damage to endothelial cells as the COVID-19 virus has an affinity for angiotensin-converting enzyme-2 (ACE2) receptors in various parts of the body like the respiratory tract, heart, gastrointestinal tract, and distal vasculature. Additionally, the activation of the tissue factor pathway, excessive thrombin generation, increased fibrin formation, and polymerization with fibrinolysis shutdown all contribute to the development of thrombosis in COVID-19 patients [1-7]. Hypoxia in severe COVID-19 infection also stimulates thrombosis by increasing blood viscosity and a hypoxia-inducible transcription factor-dependent signaling pathway [8-10,11]. The management approach is different from center to center with some institutes adopting anticoagulation protocol based on D- dimer levels and others selecting pharmacological thromboprophylaxis, preferably with intravenous unfractionated heparin or low molecular weight heparin [8-13,14-18]. Healthcare providers should have a high index of suspicion regarding this life-threatening complication of COVID-19 infection so that timely intervention can be done [14]. Also, future research is needed to better understand the role of coagulopathy and anticoagulation treatment in managing patients with COVID-19 infection [15]. 

## Case presentation

A 47-year-old male with a medical history significant for hypothyroidism, chronic kidney disease stage G2, and obesity presented to the emergency department with generalized malaise, fever, chills, watery diarrhea, weakness, body aches, productive cough of yellow sputum and shortness of breath that started nine days after he received his first COVID-19 vaccination dose. There were no associated exacerbating or alleviating factors, no exposure to new medications or change in baseline dosing of pre-existing medications, and no recent travel, exposures, or insect bites prior to the onset of his symptoms. The patient denied smoking or alcohol use. Family history was not remarkable for any significant disease.

**Figure 1:**
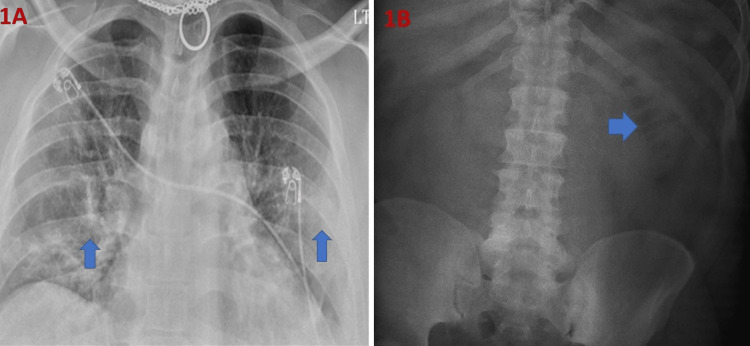
(A): Portable chest X-ray anteroposterior view demonstrated hazy patchy bilateral infiltrates (arrow) (B): Portable X-ray of abdomen supine view demonstrating overall paucity of bowel gas with questionable thickened folds in a partially distended loop of bowel in the left upper abdomen (arrow).

On presentation, the patient was noted to have sinus tachycardia (119 beats per minute), blood pressure of 112/80 mm Hg, elevated temperature of 39.4, and low oxygen saturation of 87% on room air. On physical examination, the patient had a dry mucous membrane, decreased bilateral breath sounds with no crackles or wheeze, and a soft non-distended, non-tender abdomen. Laboratory diagnostics showed elevated acute phase inflammatory markers, D-dimer, liver enzymes, serum potassium level, and creatinine (baseline creatinine: 1.5 mg/dL) (Table 1). Lactate was negative, however, COVID-19 infection came out to be positive. His chest X-ray showed bilateral hazy patchy infiltrates concerning for multilobar pneumonia (Figure 1). Electrocardiogram showed sinus tachycardia. Blood cultures were sent. 

**Table 1:** Laboratory investigations. ESR: erythrocyte sedimentation rate; CRP: C-reactive protein; AST: aspartate aminotransferase; ALT: alanine transaminase

Parameters	Normal range	Laboratory results
Creatinine	0.50 - 0.80 mg/dl	2.7 mg/dL
Potassium	3.4 -4.5 mmol/L	4.9 mmol/L
ESR	0 – 30 mm/hr	35 mm/hr
CRP	0 – 10 mg/L	60 mg/L
D- dimer	<0.5 mg/dL	20 mg/dL
AST	0 –33 units/L	58 units/L
ALT	10 – 49 units/L	64 units/L
Alkaline Phosphatase	46 – 116 units/L	120 units/L
Total bilirubin	0.3 - 1.2 mg/ dl	1.1 mg/dl
Lactic acid	0.5 - 1 mmol/L	2.2 mmol/L

The patient was started on intravenous dexamethasone 6 mg daily, intravenous fluids for rehydration, nebulization with albuterol and ipratropium, and supplemental oxygen via nasal cannula. Intravenous enoxaparin 40 mg once daily was started for deep vein thrombosis (DVT) prophylaxis. He was diaphoretic and continued to require a higher amount of oxygen; he was switched to a high-flow nasal cannula and was later transferred to the ICU. Given his increasing oxygen requirement, the intravenous dexamethasone dose was increased to 10 mg daily and baricitinib was also started. He was a candidate for remdesivir as he had been symptomatic for more than five days and his liver enzymes were mildly elevated. His repeat inflammatory markers and chest X-ray continued to worsen. A trial of prone therapy was started which seemed to help but he was unable to stay in the prone position while sleeping. 

Blood cultures came out to be negative, however, on the 5th day of admission, an empiric antibiotic for bacterial pneumonia was started along with an increment in the dose of intravenous dexamethasone to 10 mg twice daily. On day eight of admission, the patient complained of mild abdominal discomfort, however, his diarrhea had subsided by that time and there was no tenderness or rebound tenderness on palpation of the abdomen. He also denied any nausea, vomiting, or blood in the stool. His X-ray of the abdomen, as seen in Figure 1B, showed an overall paucity of bowel gas with questionable thickened folds in a partially distended loop of bowel in the left upper abdomen. He was not tolerating bilevel positive airway pressure (BIPAP) overnight and required intubation due to increased respiratory distress. He was also persistently hypotensive with mean arterial pressure in the lower 50s and was started on norepinephrine. His worsening creatinine was thought to be due to COVID-induced acute tubular necrosis as no other culprit was found. He was switched from enoxaparin to heparin for DVT prophylaxis given his worsening creatinine clearance. A temporary dialysis catheter was placed to start hemodialysis. 

**Figure 2:**
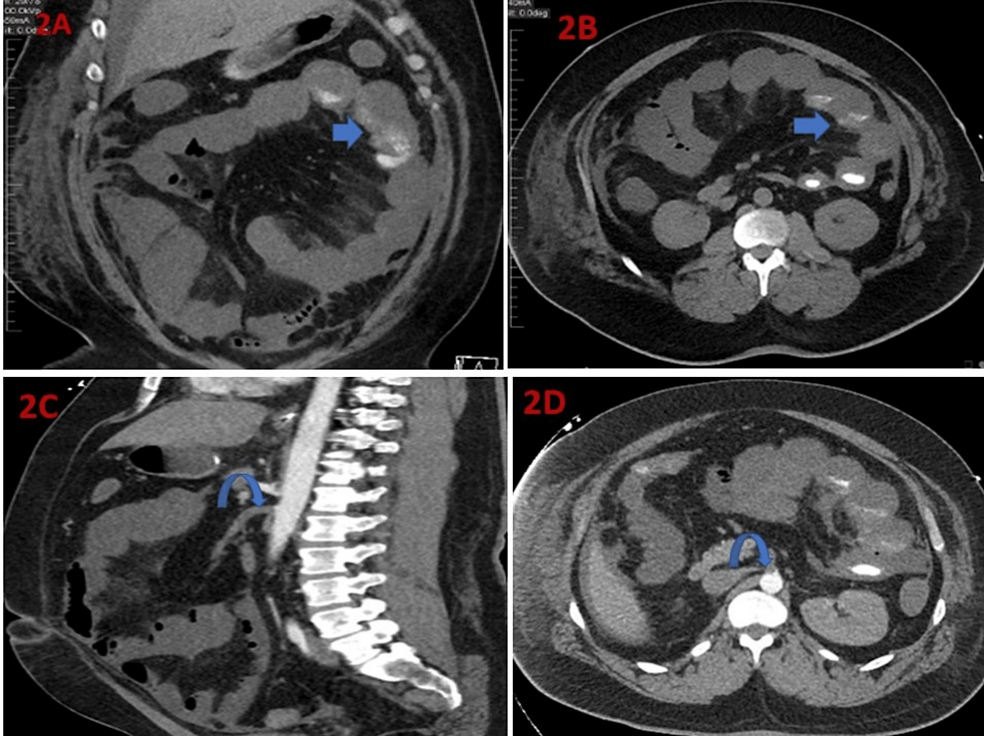
2A-2B: Non-contrast computed tomography scan of the abdomen; axial (A), and coronal (B) images of abnormal appearing mildly prominent and fluid-filled small bowel loops starting from the duodeno-jejunal junction to the fluid-filled colonic loops (arrow) with cutoff at the splenic flexure, mesenteric edema, and interloop ascites. 2C-2D: Computed tomography angiography of the abdomen; axial (C), and sagittal (D) images demonstrated: thrombus with complete occlusion of the superior mesenteric artery at its origin.

On the 12th day of the admission, there was a sudden drop in his hemoglobin level from 12.8 gm/dL to 8 gm/dL after which heparin was kept on hold. Due to the finding of a suspicious thickened fold in the bowel loops and a sudden drop in hemoglobin, a non-contrast CT of the abdomen and pelvis was performed to look for any intraabdominal/ retroperitoneal bleeding (Figures 2A-2B). CT abdomen and pelvis demonstrated abnormal appearing mildly prominent and fluid-filled small bowel loops starting from the duodenojejunal junction to the fluid-filled colonic loops with cutoff at the splenic flexure, mesenteric edema, and interloop ascites, findings concerning for ischemia, inflammation, or obstruction. Computed tomography angiography (CTA) of the abdomen demonstrated complete occlusion of the superior mesenteric artery at its origin with no evidence of atherosclerotic disease in either the superior mesenteric artery or aorta and its other branches (Figures 2C-2D). In the absence of prior thrombotic and coagulation abnormalities with no other cardiovascular risk factors promoting a hypercoagulable state, the superior mesenteric artery, and thrombus was thought to be related to the coexistent COVID-19 infection. 

The patient was emergently taken to the operating room for laparotomy. Intraoperative findings demonstrated a gangrenous small bowel extending from the ligament of Treitz to the ileocecal junction as well as extensive involvement of the cecum, ascending colon, and mid-transverse colon. Attempt at revascularization of the superior mesenteric artery and/or excision of the vast majority of the patient's bowel was not performed as it would not significantly change his outcome and quality of life. He remained intubated and was transferred back to his intensive care unit (ICU) room. The patient expired the next morning of surgery.

## Discussion

It is well known that COVID-19-associated coagulopathies favor a prothrombotic state [1,2]. Amongst patients hospitalized with COVID-19 infection, cases of both venous and arterial thromboses have been reported, with the former occurring at greater incidence [3]. The consequent sequelae of thromboembolism have been correlated to significantly elevated rates of morbidity and mortality [4]. In fact, postmortem findings from COVID-19 patients have revealed high incidences of disseminated microvascular thrombosis, with lung involvement found to be the direct cause of death in one-third of cases [5]. The incidence of intestinal thrombi-induced-ischemia, however, is a rare manifestation of COVID-19 [6].

Pathogenesis of thromboembolic events in COVID-19 infection could be better understood from the analysis of Virchow’s triad: intravascular vessel wall injury, stasis of flow, and hypercoagulable state [7]. Accumulating evidence has suggested local endothelial injury through pro-inflammatory responses and direct viral invasion through angiotensin-converting enzyme -2 (ACE2) receptors [8-10]. The surge of circulating inflammatory cytokines can increase blood viscosity and together with potential immobilization from acute illness; stasis of blood flow can result [7,11]. Finally, amplified expression of tissue factor pathways culminating in thrombin and fibrin formation, as well as hypoxia-induced platelet aggregation can produce a hypercoagulable state [7,12]. Individuals with co-existing chronic disease, obesity, and advanced age are identified as high risk for meeting these components of Virchow’s triad and provoking thrombogenesis [6]. In our case, the patient's comorbidities like chronic kidney disease and obesity along with COVID-19 infection might have evoked a hypercoagulable state.

Patients with COVID-19-induced mesenteric ischemia often share non-specific features common to other viral illnesses but gastrointestinal symptoms including diarrhea, nausea, vomiting, and abdominal pain may raise the index of suspicion [6]. Given the clinical ambiguity, repeat monitoring of D-dimer and platelet counts in addition to the use of thrombus scoring systems (i.e., Autar, Caprini, Padua, Improve) can be beneficial for thrombosis risk assessment, especially in selected patients with coagulation abnormalities on initial laboratory results [1,11]. On the other hand, abdominal imaging showcasing thrombosis, mesenteric ischemia, bowel wall thickening or pneumatosis, enlarged fluid‐filled colon, and/or pneumoperitoneum, can be more specific [13].

In our case, the clinical clues of superior mesenteric artery thrombosis were the history of diarrhea and mildly elevated D-dimer on presentation. Since the patient's hemoglobin level on presentation was at baseline that significantly dropped during the course of hospitalization, it is hard to say whether he had superior mesenteric artery thrombosis on presentation or developed thrombosis during his hospital stay. The diagnosis was only established after a follow-up abdominal X-ray and CT scan of the abdomen and pelvis which was further confirmed by CTA of the abdomen and pelvis. The patient was however provided with prophylactic enoxaparin as a part of the anticoagulation protocol for all in-patient COVID-19 patients. Indeed, the use of prophylactic anticoagulation in these patients population is currently within clinical practice guidelines [14-16]. Specifically, the use of intravenous unfractionated heparin or low-molecular-weight heparin for therapeutic and preventative use has been recommended [15,17,18]. If recognized early, management of intestinal ischemia may also require surgical or endoscopic modalities, and supportive measures like gastrointestinal decompression, fluid resuscitation, and hemodynamic support [13]. Unfortunately, our case of intestinal ischemia was severe at the time of diagnosis, and the patient was not an eligible candidate for such management strategies.

## Conclusions

Given that COVID-19 bears a significant thrombotic risk with fatal consequences, clinicians are urged to critically evaluate the combination of clinical symptoms and initial laboratory results for possible intestinal thrombosis, warranting further workup with diagnostic imaging. Future research is needed to help guide early diagnosis and the timing of initiation, dose, and duration of anticoagulation therapy in the management of patients with COVID-19 infection.
